# Formulation Optimization and Assessment of Dexamethasone Orally Disintegrating Tablets Using Box-Behnken Design

**Published:** 2018

**Authors:** Hadis Soroush, Fatemeh Ghorbani-Bidkorbeh, Seyed Alireza Mortazavi, Ali Mehramizi

**Affiliations:** a *Department of Pharmaceutics, Faculty of Pharmacy, Islamic Azad University, Pharmaceutical Sciences Branch (IAUPS), Tehran, Iran. *; b *Department of Pharmaceutics, School of Pharmacy, Shahid Beheshti University of Medical Sciences, Tehran, Iran. *; c *Tehran Chemie Pharmaceutical Co., Tehran, Iran.*

**Keywords:** Orally disintegrating tablet, Design of experiment, Dexamethasone, Optimization

## Abstract

The aim of this study was to prepare orally disintegrating tablets (ODTs) containing dexamethasone (DEX) by direct compression method with sufficient hardness and rapid disintegration time. In order to save time, money, and human resources in designing and improvement of formulation, the statistical software Design Expert is used. Box–Behnken response surface methodology was applied to evaluate and optimize the effects of concentrations of three excipients, Kollidon CL-SF (X1), Pearlitol SD200 (X2), and Prosolv SMCC (X3) as independent factors on four responses: percentage of drug released after 5 min, disintegrating time, hardness, and friability. Thirteen formulations offered by the Box–Behnken design were prepared by direct compression method and ultimate weight of 200 mg, while the amount of DEX was 4 mg. All formulations were characterized for parameters such as diameter, hardness, weight, thickness, friability, and disintegration time. Following the statistical results, the effects of independent variables on responses were evaluated and the optimum formulation regarding acceptable responses consisted of 15% Kollidon, 39.66% Pearlitol, and 7.5% Prosolv which showed 95.28% release of the drug after 5 min, disintegrating time of 30 sec, 6.1 kg hardness, and 0.12% of friability with an acceptable taste as the optimized formulation.

## Introduction

Oral administration is one of the most important and extensively used methods of using drugs with systemic effectiveness, preferred to other administrations. Furthermore, solid form among different dosage forms has the upper hand, due to high stability, easy transportation and high precision in administration. However, one of the problems with solid form is dysphagia, which is more common among children, the elderly, and other individuals with nausea and vomiting, aphthous stomatitis due to chemotherapy, Parkinson disease, motion sickness, lack of consciousness, and mental disability (1-4). ODTs are considered as one of the novel solid dosage forms which turn immediately into liquid in less than a minute and release their drug into mouth after taking into mouth and touching saliva. These tablets have had enormous improvement in recent years due to high patient compliance and ease of administration. ODTs have the benefits of solid dosage forms and after taking into mouth have the benefits of liquid dosage forms. Moreover, considering pregastric absorption of the drug, reduction of first pass metabolism, rapid onset of action, and higher bioavailability are expected (5-7). 

Dexamethasone (DEX) is one of the potent synthetic analogs and systemic cortisol which is practically aqueous insoluble (8-10). Choosing DEX as a glucocorticoid is due to its current consumption in the prevention and treatment of nausea and vomiting after chemotherapy, treatment of inflammation, and pain in gums after dental surgery, and also treatment of asthma, bronchitis, and acute croup in children (11-17), in which the patient does not respond well to the swallowing of the drug. 

Different techniques are used to manufacture ODTs, among which direct compression method is utilized in this study. In spite of simplicity and low costs, this method is strongly influenced by the properties of materials used in the integration of powder. Compromising between tablet hardness and disintegration time in ODTs prepared by direct compression method has always been a challenge and great efforts have been done by most researchers to overcome. Therefore, specific excipients are needed for direct compression method in ODTs production (18, 19). One of the widely used methods of manufacturing excipients is co-processing. The most significant property of these excipients is reduction of component separation during the process by optimizing the flow and compressibility, which results in a less variation of tablet weight, less lubricant need, lower disintegration time, and proper tablet hardness. On the other hand, these excipients are suitable choices for increasing dissolution rate of insoluble drugs (20). Furthermore, applying design of the experiment by the detection of effective factors in the experiment and determining different levels of the factors with minimum number of experiments reaches the expected results (21, 22). Singh *et al*. used experimental design to design and optimize the ODT of lamotrigine and selected the levels of disintegrant, lubricant, and tablet hardness as independent variables, and disintegration time, friability, and the drug release as responses (23).

The aim of this study is to improve properties of Orally Disintegrating Tablets produced by direct compression method such as tablet hardness, friability, disintegration time, and dissolution rate utilizing the co-processed excipients. In order to design and optimize the formulations, Box–Behnken Response-Surface Methodology (RSM), was chosen while the effects of three independent factors including Kollidon CL-SF, Prosolv SMCC90 and Pearlitol SD200 concentrations were assessed on four parameters such as dissolution rate, disintegration time, tablet hardness, and friability. 

## Experimental

*Materials*

All following materials were obtained as gift samples from Tehran Chemie Pharmaceutical Co., Tehran, Iran: Dexamethasone (Alborz Bulk Pharmaceutical Co., Iran), Crospovidone (Kollidon CL-SF, BASF, Germany), Mannitol (Pearlitol SD200, Roquette America, USA), Prosolv silicified microcrystalline cellulose (Prosolv SMCC90 JRS,USA), Lactose (Pharmatose DC14 DMV international, Holand), Magnesium stearate (St-Mg), Sodium chloride, Citric acid, flavours (Kerpen, Germany), Saccharin sodium and Aspartame. All other chemicals and solvents used were of analytical grade.

*Methods*

*Experimental Design*

Design of experiment with the minimum number of experiments in preparing formulations with different variables comes to save time and costs. Methods used in experimental design include: mixture, factorial, combined, and response surface (21). In this study Box-Behnken response surface methodology as a model of Design Expert Software (Version 7, Stat- Ease Inc., Minneapolis, MN) for optimizing ODTs of DEX and assessment of data is used. After basic studies to choose effective factors and determining levels of each factor, the independent factors were concentrations of Kollidon CL-SF (X_1_), Pearlitol SD200 (X_2_), and Prosolv SMCC (X_3_), each were studied at three levels. Table 1 shows the domain of each factor. The effects of these three factors were assessed for four responses, including: percentage of drug released after 5 min (Y_1_), disintegrating time (Y_2_), hardness (Y_3_), and friability (Y_4_) – used as dependent variables. The highest and the lowest level of each factor are coded as 1 and -1 respectively and 0 is the mean value. The central point is repeated three times to estimate experiments errors. Each of dependent variables can be shown with one polynomial equation:

$Y=B_{0}+B_{1}X_{1}+B_{2}X_{2}+B_{3}X_{3}+B_{12}X_{1}X_{2}+B_{23}X_{2}X_{3}+B_{13}X_{1}X_{3}+B_{11}X_{1}^{2}+B_{22}X_{2}^{2}+B_{33}X_{3}^{2}$                            (1)

Y = measured response, B_0 _= intercept, B_1_-B_33 _= regression coefficients for the factors and X_1_, X_2_ and X_3 _= independent factors, X_i_X_j _= the interaction terms, and X^2^_i _(i = 1, 2 or 3) are the quadratic terms. Value of factors reflects the effect of dependent variables. At each stage, the multiple correlation coefficient (R^2^) was calculated to show the model accuracy. Positive (+) and Negative (–) coefficients show the synergistic effect and the antagonistic effect, respectively. Statistical significance test of each effect is studied with ANOVA in which, if the *P*-value is <0.05, the effect is signiﬁcant. Finally, in order to illustrate the relationship between the different experimental variables and the responses, contour plots and response surface 3D plots were generated (21, 23-29).

**Table 1:** The Dependent and Independent Variables Used in Box-Behnken design to optimize the formulation

	**Levels**		
	**Low (−1) (%)**	**Middle (0) (%)**	**High (1) (%)**
Independent variable, factor X 1: Kollidon CL-SF concentration	5	10	15
X 2: Pearlitol SD200 concentration	25	35	45
X 3: Prosolv SMCC concentration	2.5	5	7.5
Dependent variable, response	Constraints		
Y 1 = drug released after 5 min (%)	Maximize		
Y 2 = disintegration time (s)	Minimize		
Y 3 = hardness (kg)	Maximize		
Y 4 = friability (kg)	Minimize		

*Formulation of Dexamethasone orally disintegrating tablets (ODTs)*

Prior to formulation preparation, physicochemical properties of DEX like organoleptic properties, bulk density, tapped density, flow, and compressibility have been studied. Bulk density (D_b_) was measured by the USP method I and tapped density (D_t_) was determined by USP method II using a tapped density tester (Aymes, Turkey). Carr’s or Compressibility Index and Hausner Ratio of DEX and powder mix which are used to compare the flow and compressibility of the powder before and after blending are measured with the following equations (10, 30):

Hausner ratio=DtDb                                                                                     (2)

$\mathrm{Compressibilty Index}=\frac{100(D_{t}-D_{b})}{D_{t}}$                                                                                      (3)

In order to prepare ODTs containing 2% (4 mg) DEX and 1% magnesium stearate with a total weight of 200 mg, direct compression method was utilized. The other components vary according to the Box-Behnken design presented in Table 2. 

**Table 2:** Dexamethasone orally disintegrating tablet formulations and their precompression parameters

**Ingredient (% Tablet weight)**								**Precompression parameters**	
**RUN NO**	**Dexamethasone** **(API)**	**Crospovidone** **Kollidon CL-SF**	**Pearlitol** **SD200**	**Prosolv** **SMCC**	**Mg Stearate**	**Lactose** **DCL14**	**Carr’s index (%)**	**Flow**	
1 2 3 4 5 6 7 8 9 10 11 12 13	2 2 2 2 2 2 2 2 2 2 2 2 2	10 15 15 5 5 5 10 10 10 5 15 10 15	25 35 25 35 45 35 45 45 35 25 45 25 35	7.5 7.5 5 7.5 5 2.5 7.5 2.5 5 5 5 2.5 2.5	1 1 1 1 1 1 1 1 1 1 1 1 1	54.5 39.5 52 49.5 42 54.5 34.5 39.5 47 62 32 59.5 44.5	16.77 16.70 23.40 9.90 7.80 10.10 8.60 9.00 10.06 10.00 9.60 23.14 21.27	Fair Fair Passable Excellent Excellent Good Excellent Excellent Good Excellent Excellent Passable Passable	

 The formulation components were weighed on a digital balance and except lubricant were sieved separately through sieve #30 mesh. After geometrical blending of components, they were put in a polyethylene bag and were mixed under proper circular movements for 10 min. Magnesium stearate after passing through sieve #60 mesh was blended with other ingredients for 1 min. The final blend was put into the compression device (rotary tableting machine, Manesty, England) and compressed into tablets using 8 mm flat die and punches. In every batch, 40 tablets were produced. After final blending and before tablet compression, flow of each formulation was evaluated according to the mentioned method, because flow of powder is important in tablet uniformity.

 Characterization of ODTs

 The physicochemical properties of different Dexamethasone ODT formulations such as appearance, diameter and thickness, uniformity of weight, and the inﬂuence of different excipients as independent variables on % drug released after 1 min, disintegration time, hardness and friability as dependent responses were investigated (31-34). 

*Assessment of diameter, thickness and weight variation*

Twenty tablets of each formulation were randomly chosen and their diameter and thickness were measured by vernier callipers. For weight variation test, 20 tablets were randomly selected from each run and separately weighed by analytical scale and the average and standard deviation were calculated. Weight variation is a proper method to measure drug content uniformity (35). Considering the weight of each is 200 mg, maximum standard deviation is, according to USP, 7.5%. Not more than two tablets should exceed this domain; nor, none of the tablets should exceed twice the allowed perimeter (36).

*Assessment of Experimental design variables*

*In-vitro dissolution studies*

ODTs Dissolution study is similar to regular tablets; except for apparatus – USP II (paddle), which is a more common and proper device for ODTs. The *In-vitro* drug release was studied using USP Apparatus Type II (Paddle) (Electrolab, TDT-08L India), in 500 mL 0.1N hydrochloric acid as medium at 100 rpm and at 37 ± 0.5 °C. At time intervals of 1, 5, 10, 20, 45 min, 5 mL of the dissolution medium was taken and to keep the balance of the sink condition, the same amount of volume from the fresh medium of dissolution – already reached to the desired temperature – was replaced. Thereafter, the samples were filtered and assayed using the (Shimadzu UV/visible 1700 spectrophotometer Japan) at 243 nm wavelength. 

*Disintegration time*

Disintegration time was carried out using disintegration test apparatus (Electrolab, India). Nine-hundred mL of distilled water at 37 ± 2 °C was used as the disintegration medium. Six tablets per batch were chosen randomly and placed in the disintegration apparatus. Disintegration time was considered when the tablets dispersed and all the tablet fragments passed through the mesh completely. This experiment was repeated in triplicates, and then the average and standard deviation were recorded.

*Hardness*

Hardness of tablets was measured using a tablet hardness tester (Type TBH220TD, Erweka, Germany). A tablet was put into the device, and the force needed to break the tablet was recorded. Twenty tablets were evaluated in each run, and the average hardness and the standard deviation were calculated.

*Friability test*

This test was operated with Friability test apparatus (Electrolab, India). Twenty tablets in each run chosen randomly and already weighed were placed in the device and rotated with the speed of 25 rpm. After 4 min of rotating in the device, the tablets were dedusted and weighed again. The percentage friability of tablets is measured using the following equation:

 % friability=initial weight g- final weight (g)initial weight (g)×100               (4)

*Complementary tests*

*Taste evaluation*

After analysing the data of experimental design, and choosing optimized formulation, complementary tests were done on the optimized formulation. Since DEX has an unpleasant taste, and due to ODT disintegration in the mouth, and to improve patient compliance, the next stage – after optimizing and choosing the optimized formulation – is masking of the unpleasant taste of the tablet. In spite of the presence of excipients like Kollidon CL-SF, and the smooth cream-like mouth feel associated with its use, and also Pearlitol 200 SD due to freshness and sweetness it creates in the mouth, adding flavours, citric acid, sodium citrate and sodium chloride in proper dosages to improve the taste of the formulation seems necessary (37, 38). Table 3 shows the type and percentage of the materials in the taste evaluation test. In order to conduct the experiment, 10 volunteers were selected from healthy people between 20-40 years old from both sexes (5 males and 5 females), and due to moral principles, they were informed about the nature of the drug and the procedure, before conducting the experiment; also, they were asked to – with patient satisfaction – wash their mouths and put the tablet on their tongue, and refuse from swallowing the tablet during the test, and after the disintegration and testing the taste with the help of visual analogue scale, rinsed their mouths with water. According to the following scale, taste scale was rated in 6 levels (0 = like extremely, 2 = like moderately, 4 = like slightly, 6 = dislike slightly, 8 = dislike moderately and 10 = dislike extremely). Volunteers scored 0 for the best taste and 10 for the worst. Therefore, F formulation series was studied for the compliance of administrator (1, 39 and 40).

**Table 3:** Selection of flavours and sweeteners

**Ingredients**	**Quantity (mg/Tab)**				
	**Flavour selection**			**Sweetener selection**	
	**F**_1_	**F**_2_	**F**_3_	**F**_4_	**F**_5_
Dexamethasone (2%)	4	4	4	4	4
Kollidon CL_SF (15%)	30	30	30	30	30
Pearlitol SD200 (39.66%)	79.32	79.32	79.32	79.32	79.32
Prosolv SMCC (7.5%)	15	15	15	15	15
Pharmatose DCL14 (26.84%)	53.68	53.68	53.68	53.68	53.68
Magnesium stearate (1%)	2	2	2	2	2
Citric acid (3%)	6	6	6	6	6
Sodium chloride (2%)	4	4	4	4	4
Orange flavour (1%)	2				
Lemon flavour (1%)		2			2
Peppermint flavour (1%)			2		
Grape flavour (1%)				2	
Saccharin (2%)	4	4	4	4	
Aspartame (2%)					4
Total (mg)	200	200	200	200	200

*Water absorption ratio*

The tablet which had already been weighed was placed on the surface of a paper folded twice into a petri dish containing 6 mL distilled water. When the tablet absorbed the water completely, was weighed again, and the absorption ratio was measured according to the equation 5 (41). 

$R=100\frac{W_{a}-W_{b}}{W_{b}}$                                                             (5)

When W_a_ and W_b _were tablet weights before and after water absorption

*Determination of drug content*

To determine *drug content* in the optimized formulation, 10 dexamethasone ODTs were selected from the optimized formulation and crushed into powder in a mortar. An equivalent of one tablet weight was transferred into a 50-mL volumetric flask, then 25 mL solution (methanol: water 1: 2 v/v) was added. The flask was shaken and sonicated for 15 min, and then the solution was diluted to required volume with the same fluid, and after filtering through filter paper the absorption ratio was read – using spectrophotometer at pre-determined λ_max_ of 241 nm. Finally, drug content in the optimized formula is measured.

## Results and Discussion

DEX is a white crystalline, colorless, and odourless powder with a slightly bitter taste and very poor flow and compressibility (9). Flow properties were assessed determining bulk density, Tapped density, Carr’s Index and Hausner Ratio. Bulk and tapped density were determined to be 0.250 ± 0.01 and 0.375 ± 0.01 g/cm^3^, respectively. Carr’s index and Hausner Ratio were achieved as 33.3% ± 1.80 and 1.5 ± 0.04, respectively. Therefore, DEX has a very poor flow and regarding the fact that flow is important in tablet uniformity, selecting excipients with proper flow, especially in direct compression, is of great significance. The results of flow in 13 ODT formulations in Table 2 shows integrating flow, due to the use of Peralitol SD200 and Prosolv SMCC as two co-processed excipients in formulation of the tablet, which was highly effective (19). Peralitol SD200 is a co-processed excipient and a spheronised spray-dried mannitol which is used for products that are prepared with direct compression method. It is also granulized, white, odourless, slightly sweet, crystallized, non-moisturizing, highly stable and with excellent flowability, compressibility, and high solubility. Prosolv SMCC, which is made of combination of 98% microcrystalline cellulose and 2% colloidal silicone dioxide, is a co-processed silicified microcrystalline cellulose, which improves the compressibility and flowability and thereby decreases the weight difference in the DC method.

The results of diameter, thickness, and tablet weight variation are mentioned in Table 4, and standard deviation in all formulations is lower than mentioned domain in United States Pharmacopoeia (USP) which indicates the integration of the drug and excipients have been all proper and homogenous as expected for DC formulations with good flow properties. Tablet diameter and thickness showed low variability supporting the reproducibility of formulations and tableting process.

**Table 4:** Evaluation of different physical parameters of dexamethasone ODTs

^Batch^	^Avg. weight ± SD n = 20^	^Thickness (mm)^ ^n = 10^	^Diameter (mm)^ ^n = 10^	^Surface^
^1^	^201.26 ± 2.36^	^4.24 ± 0.02^	^8 ± 0.005^	^Smooth^
^2^	^202 ± 1.24^	^4.36 ± 0.01^	^8.05 ± 0.001^	^Smooth^
^3^	^202.6 ± 1.95^	^4.43 ± 0.03^	^8.07 ± 0.001^	^Smooth^
^4^	^202.8 ± 1.39^	^4.20 ± 0.01^	^8 ± 0.010^	^Smooth^
^5^	^200.5 ± 0.53^	^4.25 ± 0.02^	^8.04 ± 0.005^	^Smooth^
^6^	^200.54 ± 0.45^	^4.20 ± 0.98^	^8.02 ± 0.035^	^Smooth^
^7^	^202.42 ± 1.51^	^4.22 ± 0.04^	^8.025 ± 0.015^	^Smooth^
^8^	^201.31 ± 0.78^	^4.30 ± 0.02^	^8.04 ± 0.010^	^Smooth^
^9^	^200.81 ± 0.85^	^4.35 ± 0.01^	^8.07 ± 0.010^	^Smooth^
^10^	^200.67 ± 0.64^	^4.30 ± 0.07^	^8.045 ± 0.007^	^Smooth^
^11^	^201.14 ± 0.82^	^4.37 ± 0.01^	^8.05 ± 0.005^	^Smooth^
^12^	^200.58 ± 0.35^	^4.41 ± 0.01^	^8.06 ± 0.005^	^Smooth^
^13^	^201.5 ± 0.52^	^4.45 ± 0.02^	^8.12 ± 0.014^	^Smooth^

Values as Mean ± SD.

*Box-Behnken design, Statistical analysis and mathematical modelling*

Designing experiments is one of the most important issues that is nowadays discussed in various industries, especially research and development activities in pharmaceutical industry. In fact, statistical design for empirical experiments is considered as a basic principle in conducting laboratory and industrial researches. Such designs lead to results with more confidence, saving time, and considerable reduction of number of experiment repetitions and eventually lead to optimization of the process. Considering the type of formulation for ODTs, response surface methodology (RSM) was chosen among the available methods in the software for designing the formulations. This method exclusively studies the relation between the response and the effective factors on the response. Regression models are used for analyzing the response. The focus of this method on specifying the nature of relation between the response and the factors is far more important than identifying such factors, as it is possible to optimize a number of possible responses at that time. The RSM method, on the other hand, is a set of mathematical and statistical techniques for design. The experiments in this method study the effects of various factors and evaluate obtaining the optimal conditions in order to achieve the desired response. The most common models of this method is BB, that is a spherical design, which due to its reasonable design and excellent results is utilized for optimization and even for other applications. BB was also chosen among the various RSM models. The advantages of this model are as following: Only three levels of each factor are studied (-1, 0, 1), when the number of variables is 3 or 4, then the total number of experiment runs is lower comparing with other models, which for a study of three factors, the total number of experiments are 13 runs and considering the repetitions of the centre point, this number will reach to 15 runs. The condition in which all variables have the maximum and minimum amount is not studied in this method and thereby excess and negligence is avoided in conducting the experiments (21).

The results of 13 offered formulations by Box–Behnken indicates that regarding *P*-value < 0.05, the offered model of the Box–Behnken response surface methodology in all four dependent variables was significant. The model in all dependent variables – except for friability which is a function of quadratic model – follows linear model according to the Analysis of variance (ANOVA). 

Statistically significant coefficients (*P* < 0.05) were only retained in the equations. The relative influence of each variable on the responses can be signified by the magnitude and sign of the main effects. The results of all four responses are summarized in Table 5. 

**Table 5:** Three-factors with measured responses of the Box-Behnken design

**Exp no.**	**X**_1 _ **(%)**	**X**_2 _ **(%)**	**X**_3 _ **(%)**	**Y**_1 _ **(%)**	**Y**_2 _ **(sec)**	**Y**_3 _ **(kg)**	**Y**_4 _ **(%)**
1	10	25	7.5	91.75	31	5.1	0.37
2	15	35	7.5	102.21	30	4.9	0.11
3	15	25	5	87.60	32	4.8	0.33
4	5	35	7.5	91.42	34	4.6	0.33
5	5	45	5	90.50	38	4.2	0.45
6	5	35	2.5	89.70	42	3.4	0.37
7	10	45	7.5	92.90	33	6.5	0.26
8	10	45	2.5	95.05	36	5.9	0.35
9	10	35	5	92.85	34	5.3	0.22
10	5	25	5	80.40	35	2.8	0.48
11	15	45	5	104.62	34	7	0.27
12	10	25	2.5	87.60	33	3.8	0.29
13	15	35	2.5	94.25	39	4.6	0.19

**Figure 1:**
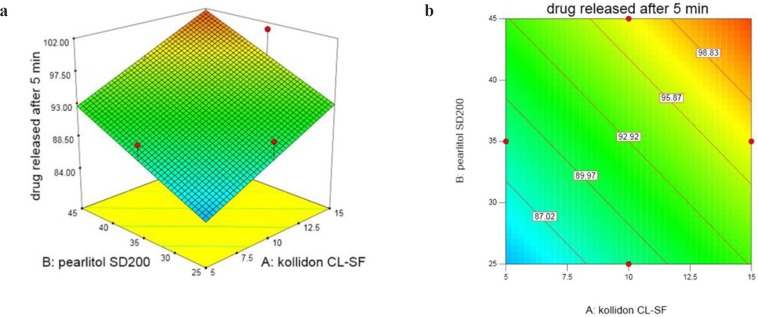
(a) Response surface and (b) Contour plot of the effect of Kollidon CL- SF(X_1_) and Pearlitol SD200 (X_2_) on % Dex released after 5 min (Prosolv Concentration = 7.5%)

Percentage of DEX released after 5 min (Y_1_) in 13 ODT formulations varies in the range 80.40%-104.63%. As regression coefﬁcients and Figure 1 show, by increasing three independent variables, the cumulative percent of the released drug also increases with a high degree of correlation. Three dimensional response surface and contour plots offer graphically the significance of regression equations and presenting minima and maxima. Different colour regions demonstrate the variation in values. ANOVA test reveals that only the effect of X_1_ and X_2 _are significant. The following equation shows the relationship between dependent variables with percentage of DEX released after 5 min (Y_1_): 

$Y_{1}=24.263+1.560X_{1}+0.766X_{2}+1.489X_{3}$                                                     (6)

**Figure 2:**
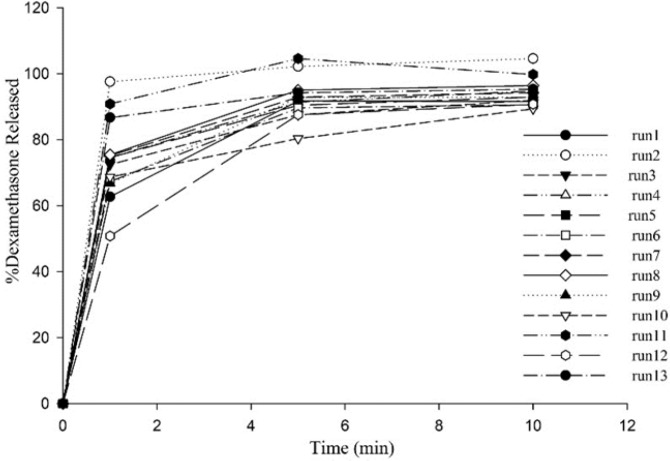
Dissolution profiles of all formulations in HCl 0.1 N

All ODTs released over 90% drug at the end of 10 min (Figure 2). X_1_ and X_2 _are Kollidon CL-SF and Pearlitol 200 SD concentrations, respectively and as can be seen in Equation 5, have positive effects on dissolution rate. Kollidon CL-SF helps dissolution process and especially improves dissolution rate of practically insoluble drugs due to narrow particle size distribution, capillary action and swelling ability of this excipient without gel formation, as well as its disintegrating effect. Pearlitol 200 SD as a co-processed excipient, with desired porous texture dissolves quickly and increases water permeability inside the tablet and thereby increases the release rate of drugs. The least percentage of drug release was for run 10 with 80.40% and the most percentage of drug release was for run 11 with 104.63%. As it is evident, the Kollidon CL-SF and Pearlitol SD200 content in run 10 are at their lowest amount and these two variables are at their highest amount in run 11.

**Figure 3:**
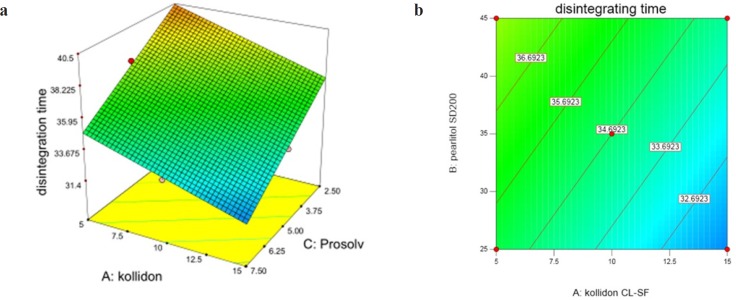
(a) Response surface and (b) Contour plot of the effect of Kollidon CL-SF (X_1_), Pearlitol SD200 (X_2_) and Prosolv SMCC (X_3_) on disintegration time of ODT

InY_2 _(disintegration time) according to variation domain 30-42 sec, two variables X_1 _and X_3 _(Prosolv concentration) have significant and antagonistic effects, while the effect of X_2 _is non-significant and synergistic. These results are shown in Figure 3. Following equation shows the relationship between dependent variables and disintegration time: 

$Y_{2}=39.317-0.350X_{1}+0.125X_{2}-1.100X_{3}$                                           (7) 

Kollidon CL-SF as a superdisintegrant is able to absorb great amounts of water exposing to the aqueous environment, furthermore the combination of swelling and water absorption results in breaking tablets and finally faster disintegration. Prosolv SMCC as a co-processed excipient consists of microcrystalline cellulose and silicon dioxide, which has a homogenous and fine particle size distribution offering a great specific surface area and decreasing disintegration time. It can be seen from the response surface plot (Figure 3) that the lowest disintegration time was achieved at 15% Kollidon and 35% Pearlitol and 7.5% Prosolv concentrations and it is clear that DT (Y_2_) is greater at lower levels of Kollidon and Prosolv (X_3_). As it is evident, the longest disintegration time was seen in run 6 containing the least Kollidon and Prosolv contents. Pearlitol is a polyol material, soluble in water and is a hexa-hydrated alcohol that competes with Kollidon for water penetration into the tablet. Disintegration of the tablet weakens upon increase of Pearlitol concentration and the effect of Kollidon is reduced.

**Figure 4.**
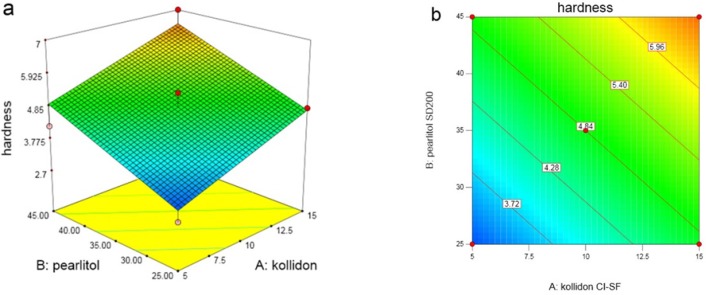
(a) Response surface and (b) Contour plot of the effects of Kollidon CL-SF (X_1_) and Pearlitol SD200 (X_2_) on ODT hardness

In Y_3_ (hardness), as shown in the Figure 4 and the variation domain 2.8–7 kg, with increasing all 3 variables, hardness also increases. This increase in X_1_ and X_2_ is significant and in X_3_ is non-significant. Following equation shows the relationship between dependent variables and hardness:

$Y_{3}=-0.692+0.157X_{1}+0.088X_{2}+0.170X_{3}$                                   (8)

Hardness of ODTs is usually considered lower than usual tablets, which is an important factor in ODTs; if the tablet is not hard enough, it damages through packing, storage, handling, or transformation. Meanwhile, the dissolution and disintegration shouldn’t have a problem due to the hardness. Pearlitol SD200 is a spray-dried mannitol which improves flow and compressibility; furthermore it increases hardness of the tablet. Kollidon CL-SF has a plasticity feature and other physical properties of Copovidone such as particle structure and size which can influence the hardness and give hard tablets. Binding properties and plasticity of this matter makes it an appropriate adherent, which as its concentration increases, tensile strength of the tablet increases as well and thereby protect the tablet against any abrasion during transportation and consumption. The lowest tablet hardness was seen in run 10 with the least amount of Kollidon and Pearlitol, and the highest tablet hardness was seen in run 11 with maximum content of these two independent variables. There is a direct relation between disintegration time and hardness of the tablet. As the hardness increases, the pores are closed and thereby water permeability is decreased which affects the disintegration time of the tablet. The highest tablet hardness was achieved at 15% Kollidon, 45% Peralitol, and 5% Prosolv concentrations. 

**Figure 5:**
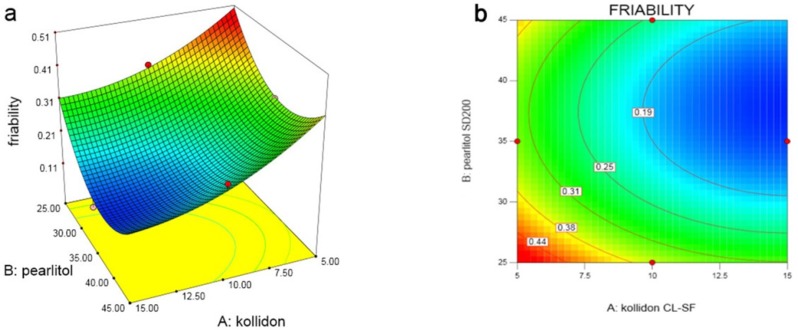
(a) Response surface and (b) Contour plot of the effect of Kollidon CL-SF (X_1_) and Pearlitol SD200 (X_2_) on ODT friability

In the last variable (Y_4_), which studies the friability of the tablet as shown in the response surface and contour plots (Figure 5), with increasing amount of Kollidon SL-CF (X_1_), friability decreases, but increasing Pearlitol SD200 (X_2_), in low level of Prosolv, friability increases and in high level, friability decreases. The same trend can be seen in X_3_ variation at low and high concentrations of X_2_. Following equation shows the relationship between dependent variables and friability:

Y4=1.635-0.047X1-0.072X2+0.089X3-1.500 E-004X1X2-8.000E-004X1X3-1.700E-003X2X3+1.900E-003X12+1.150E-003X22-2.800E-003X32                     (9)

As the analysis results indicates, the highest friability was seen in run 10 with the minimum content of all three variables, and the least friability was seen in run 2 with maximum concentration of Kollidon and Prosolv. Pearlitol, at a moderate concentration of 35% have less friability due to its crystalline and porous structure, while Kollidon with its high compressibility, reduced friability at high concentrations.

According to the results of the analysis, although it was observed that the effect of Prosolv SMCC on the three dependent variables; drug release percent after 5 min, hardness, and friability is insignificant and is only significant concerning the disintegration time, for direct compression method of tablets it is important to select the proper pharmaceutical excipients to achieve a good compressibility. Prosolv has a better flowability and compressibility in comparison to microcrystalline cellulose and colloidal silicone dioxide and physical mixture of the two ingredients. In the other words, it is more compressible than normal cellulose and furthermore increases uniformity of the content and the adaptation of the active ingredient with other excipients, and also decreases the disintegrating time of the tablet. Prosolv slightly increases the hardness of the tablet, reduces friability and avoids deformation of the particles during the tableting process (4, 19).

*Optimization*

According to the results, for each response in Table 6 after analyzing the data and determining the domain for each dependent and independent variable in numerical optimization section, the plot contains Kollidon 15%, Pearlitol 39.66%, and Prosolv 7.5% with 0.95 desirability which was chosen as the optimized formulation. The optimum formulation was prepared and characterized as done for 13 formulations. The results were compared with the predicted results, calculating residuals, and prediction error% (Table 6) in order to show the validity of Box-Behnken Design formulation suggestion. The optimized formula was further evaluated in complementary tests of taste, water absorption, and determination of drug content.

**Table 6:** The suggested optimum formula with predicted and observed responses, residuals and prediction error%

**Independent variable**	**Optimum**	**Dependent variable**	**Predicted**	**Observed**	**Residuals**	**Prediction Error (%)**
X_1_ (%)	15	Y_1 _(%)	91.46	95.28	-3.82	-5.42
X_2_	39.66	Y_2_ (sec)	30.77	30.00	0.77	2.51
X_3_	7.5	Y_3_ (kg)	6.46	6.10	0.36	5.57
		Y_4_ (%)	0.13	0.12	0.01	7.69

Finally, comparing the volunteers’ results, among 10 formulations, F5 containing: NaCl 2%, lemon flavor 1%, aspartame 2% and citric acid 3% was chosen as the final formulation. It should be mentioned that citric acid as saliva stimulating agent, reduces durability of the tablet in mouth by increasing the secretion of saliva, and stimulates it for easier administration. Also, sodium chloride helps the unpleasant after taste (42, 43). The result of drug content determination in selected formulation was 98.07% ± 0.05 in methanol: water (1:2) medium. Water absorption ratio in the optimized formulation was also 113.5% ± 2. 

## Conclusion

ODTs have more patient compliance than the regular tablets and have the ability to defeat all the problems of swallowing. In this study DEX ODT was prepared with high release of drug at the end of 5 min, disintegration time less than a minute, proper hardness, friability, and pleasant taste. This study demonstrated that applying co-processed directly compressible excipients in producing ODTs containing insoluble drugs with low flowability like DEX can improve compressibility, increase drug release and hardness, and it can also decrease disintegration time and friability. On the other hand, applying BB design in the evaluation of excipients in optimizing the ODT formulation was effective and the results of conducting the experiment for optimization in all 4 expected responses by the experimental design were very close and in-line, which shows its application in DEX ODTs production is more effective and economical. 

## References

[1] Tayebi H, Mortazavi SA (2011). Formulation and evaluation of a novel matrix-type orally disintegrating ibuprofen tablet. Iran. J. Pharm. Res.

[2] Pallikonda AL, Bairam R, Motilal M, Kumar MS (2010). Formulation and evaluation of mouth dissolving tablets. Sch. Res. Lib.

[3] Sharma D (2013). Formulation development and evaluation of fast disintegrating tablets of salbutamol sulphate for respiratory disorders. ISRN Pharm.

[4] Singh J, Philip AK, Pathak K (2008). Optimization studies on design and evaluation of orodispersible pediatric formulation of indomethacin. AAPS PharmSciTech.

[5] Ghosh TK, Pfister WR (2005). Drug Delivery to the Oral Cavity: Molecules to Market.

[6] Parkash V, Maan S, Deepika SKY, Hemlata VJ (2011). Fast disintegrating tablets: Opportunity in drug delivery system. J. Adv. Pharm. Technol. Res.

[7] Aguilar-Diaz JE, Garcia-Montoya E, Perez-Lozano P, Sune-Negre JM, Minarro M, Tico JR (2014). SeDeM expert system a new innovator tool to develop pharmaceutical forms. Drug Dev. Ind. Pharm.

[8] (2014). British Pharmacopoeia.

[9] Moffat AC, Osselton MD, Widdop B, Galichet LV (2011). Clarke’s Analysis of Drugs and Poisons In Pharmaceuticals, Body Fluids and Postmortem Material.

[10] (2015). The United States Pharmacopeia 38/National Formulary 33.

[11] Schuh S, Coates AL, Binnie R, Allin T, Goia C, Corey M, Dick PT (2002). Efficacy of oral dexamethasone in outpatients with acute bronchiolitis. J. Pediatr.

[12] Sweetman SC (2011). Martindale: The Complete Drug Reference.

[13] Sparrow A, Geelhoed G (2006). Prednisolone versus dexamethasone in croup: A randomised equivalence trial. Arch. Dis. Child.

[14] Shimoda H, Taniguchi K, Nishimura M, Matsuura K, Tsukioka T, Yamashita H, Inagaki N, Hirano K, Yamamoto M, Kinosada Y, Itoh Y (2009). Preparation of a fast dissolving oral thin film containing dexamethasone: A possible application to antiemesis during cancer chemotherapy. Eur. J. Pharm. Biopharm.

[15] Cronin J, Kennedy U, McCoy S, An Fhaili SN, Crispino-O’Connell G, Hayden J, Wakai A, Walsh S and O′Sullivan R (2012). Single dose oral dexamethasone versus multi-dose prednisolone in the treatment of acute exacerbations of asthma in children who attend the emergency department: Study protocol for a randomized controlled trial. Trials.

[16] Cross KP, Paul RI, Goldman RD (2011). Single-dose dexamethasone for mild-to-moderate asthma exacerbations effective, easy, and acceptable. Can. Fam. Physician.

[17] Dollery CT (1999). Therapeutic Drugs.

[18] Late SG, Yu YY, Banga AK (2009). Effects of disintegration-promoting agent, lubricants and moisture treatment on optimized fast disintegrating tablets. Int. J. Pharm.

[19] Chowdary K, Ramya K (2013). Recent research on co-processed excipients for direct compression-A review. Int. J. Comprehensive Pharm.

[20] Schiermeier S, Schmidt PC (2002). Fast dispersible ibuprofen tablets. Eur. J. Pharm. Sci.

[21] Armstrong NA (2006). Pharmaceutical Experimental Design and Interpretation, 2nd ed.

[22] Bolourchian N, Hadidi N, Foroutan SM, Shafaghi B (2008). Formulation and optimization of captopril sublingual tablet using D-Optimal design. Iran. J. Pharm. Res.

[23] Singh J, Garg R, Gupta GD (2015). Enhancement of solubility of lamotrigine by solid dispersion and development of orally disintegrating tablets using 32 full factorial design. J. Pharmaceutics.

[24] Tung NT, Hung MV, Vo XM, Nguyen TH (2014). Formulation optimization of orally disintegrating tablets containing solid dispersion of felodipine and hydroxypropyl methylcellulose using face-centered central composite design. J. Pharm. Investig.

[25] Hayashi Y, Oshima E, Maeda J, Onuki Y, Obata Y, Takayama K (2012). Latent structure analysis of the process variables and pharmaceutical responses of an orally disintegrating tablet. Chem. Pharm. Bull.

[26] Butte K, Momin M, Deshmukh H (2014). optimisation and in-vivo evaluation of pectin based drug delivery system containing curcumin for colon. Int. J. Biomater.

[27] Ibrahim HK, El-Setouhy DA (2010). Valsartan orodispersible tablets: Formulation, in-vitro/in-vivo characterization. AAPS PharmSciTech.

[28] Bolourchian N, Hadidi N, Foroutan SM, Shafaghi B (2009). Development and optimization of a sublingual tablet formulation for physostigmine salicylate. Acta Pharm.

[29] Sammour OA, Hammad MA, Megrab NA, Zidan AS (2006). Formulation and optimization of mouth dissolve tablets containing rofecoxib solid dispersion. AAPS PharmSciTech.

[30] Aulton ME (2002). Pharmaceutics: The Science of Dosage Form Design.

[31] Niazi SK (2009). Handbook of Pharmaceutical Manufacturing Formulations: Sterile Products.

[32] Patra S, Sahoo R, Panda R, Himasankar K, Barik B (2010). In-vitro evaluation of domperidone mouth dissolving tablets. ‎Indian J. Pharm. Sci.

[33] Florence AT, Siepmann J (2009). Modern Pharmaceutics. Basic Principles and Systems.

[34] Remington JP, Allen LVJr (2006). Remington: The Science and Practice of Pharmacy.

[35] Harada T, Narazaki R, Nagira S, Ohwaki T, Aoki S, Iwamoto K (2006). Evaluation of the disintegration properties of commercial famotidine 20 mg orally disintegrating tablets using a simple new test and human sensory test. Chem. Pharm. Bull.

[36] Allen LV, Popovich NG, Ansel HC (2013). Ansel’s Pharmaceutical Dosage Forms and Drug Delivery Systems.

[37] Patil P, Shrivastava S (2014). Formulation, evaluation and optimization of fast dissolving oral film of selective antihypertensive drug. World J. Pharm. Sci.

[38] Douroumis D (2011). Orally disintegrating dosage forms and taste-masking technologies. Expert Opin. Drug Deliv.

[39] Reza HM, Islam T, Shohel M, Jain P (2012). Formulation development of taste masked aceclofenac orodispersible tablets. American-Eurasian J. Sci. Res.

[40] Vummaneni V, Nagpal D (2012). Taste masking technologies: An overview and recent updates. Int. J. Res. Pharm. Biomed. Sci.

[41] Abdelbary A, Elshafeey A, Zidan G (2009). Comparative effects of different cellulosic-based directly compressed orodispersable tablets on oral bioavailability of famotidine. Carbohydr. Polym.

[42] Sharma S, Lewis S (2010). Taste masking technologies: A review. Int. J. Pharm. Pharm. Sci.

[43] Sheshala R, Khan N, Darwis Y (2011). Formulation and optimization of orally disintegrating tablets of sumatriptan succinate. Chem. Pharm. Bull.

